# Cost-effectiveness analysis of inavolisib combined with palbociclib plus fulvestrant in PIK3CA-mutated HR+/Her2− advanced breast cancer in China

**DOI:** 10.3389/fphar.2025.1742507

**Published:** 2026-01-05

**Authors:** Weiwei Huang, Cijuan Li, Yuanqing Huang, Chang Wang, Yingxin Zhang, Yingtao Lin

**Affiliations:** 1 Department of Medical Oncology, Clinical Oncology School of Fujian Medical University, Fujian Cancer Hospital, Fuzhou, China; 2 Fujian Provincial Key Laboratory of Cancer Metastasis, Cancer Metastasis Alert and Prevention Center, College of Chemistry, Fuzhou University, Fuzhou, Fujian, China; 3 Department of Comprehensive Surgery, Fujian Maternity and Child Health Hospital, College of Clinical Medicine for Obstetrics & Gynecology and Pediatrics, Fujian Medical University, Fuzhou, China; 4 Hospital Vice-Superintendent, Fujian Maternity and Child Health Hospital, College of Clinical Medicine for Obstetrics & Gynecology and Pediatrics, Fujian Medical University, Fuzhou, China; 5 Department of Lymphoma & Head and Neck Tumors, Clinical Oncology School of Fujian Medical University, Fujian Cancer Hospital, Fuzhou, China; 6 Clinical Medical Research Center, Clinical Oncology School of Fujian Medical University, Fujian Cancer Hospital, Fuzhou, China

**Keywords:** breast cancer, cost-effectiveness analysis, inavolisib, partitioned survival model, PIK3CA

## Abstract

**Background:**

Inavolisib, a selective PI3Kα inhibitor, combined with palbociclib and fulvestrant, has demonstrated significant clinical benefit in PIK3CA-mutated hormone receptor–positive/human epidermal growth factor receptor 2–negative (HR+/HER2−) advanced or metastatic breast cancer (ABC/MBC). However, its cost-effectiveness in China remains unclear.

**Methods:**

A partitioned survival model with three health states—progression-free survival (PFS), progressive disease (PD), and death—was developed to evaluate inavolisib plus palbociclib-fulvestrant versus palbociclib-fulvestrant alone from the Chinese healthcare perspective. Survival data were derived from the phase III INAVO120 trial, while costs and utility values were obtained from local sources and literature. The model estimated total costs, quality-adjusted life years (QALYs), and incremental cost-effectiveness ratios (ICERs). Deterministic and probabilistic sensitivity analyses (DSA and PSA) and scenario analyses were conducted to assess model robustness and the impact of drug price and time horizon variations.

**Results:**

Inavolisib combination therapy increased total costs ($194306.06 vs. $55938.19) and QALYs (2.999 vs. 1.744), resulting in an ICER of $110260.53/QALY, exceeding the Chinese willingness-to-pay (WTP) threshold of $40271.00/QALY. ICER was most sensitive to PFS utility and drug cost. Scenario analyses indicated that inavolisib would become cost-effective if its price decreased by approximately 88.53%.

**Conclusion:**

While inavolisib plus palbociclib-fulvestrant significantly prolongs PFS and OS in PIK3CA-mutated HR+/HER2− ABC/MBC, it is not cost-effective at current prices in China. Strategic price adjustments or reimbursement negotiations are essential to improve economic feasibility and inform clinical and policy decisions.

## 1 Introduction

Breast cancer is the most frequently diagnosed malignancy in women and ranks second among all cancers worldwide in terms of incidence (Bray et al., 2024). According to data from the World Health Organization, an estimated 2.3 million women were newly diagnosed with breast cancer in 2022, resulting in approximately 670,000 deaths (World Healeth Organization, 2025). In China, 357,161 new cases were reported in the same year, with 74,986 associated deaths (World Health Organization, 2024). Clinically, breast cancer is classified into three major subtypes: hormone receptor (HR)-positive/human epidermal growth factor receptor 2 (HER2)-negative (HR+/HER2−), HER2− positive (HER2+; defined by gene amplification or receptor overexpression), and triple-negative (HR−/HER2−). Among these, the HR+/HER2− subtype is the most prevalent, accounting for approximately 65%–70% of all breast cancer cases (Serra et al., 2019). Owing to its high incidence and mortality, breast cancer imposes a substantial economic burden and represents a major public health challenge globally and in China.

Endocrine therapy in combination with cyclin-dependent kinase 4/6 (CDK4/6) inhibitors is currently the standard first-line treatment for patients with HR+/HER2− advanced breast cancer (ABC) (China Anti-cancer Association Committee of Breast Cancer Society, 2025; National Quality Control Center for Cancer Breast Cancer Expert Committee, 2023). However, the development of drug resistance remains an inevitable clinical challenge. Increasing evidence indicates that abnormalities in the phosphatidylinositol 3-kinase (PI3K)/protein kinase B (AKT)/mammalian target of rapamycin (mTOR) signaling pathway represent a major mechanism driving resistance to endocrine therapy. Although the PI3K pathway plays essential roles in both normal and malignant cells, its inhibition inevitably results in adverse effects on normal tissues (Huang et al., 2022). Activating mutations in the PI3K catalytic subunit alpha (PIK3CA) are the most common genetic alteration in HR+ breast cancer, present in approximately 35% of metastatic cases (Martínez-Sáez et al., 2020), and are strongly associated with poor prognosis (Zardavas et al., 2018). Consequently, precision therapies targeting PIK3CA mutations have emerged as a promising strategy to overcome therapeutic resistance, prolong progression-free survival (PFS), and improve overall survival (OS).

Inavolisib (GDC-0077) is a next-generation, highly selective PI3Kα inhibitor that binds to the ATP-binding site of PI3Kα, thereby inhibiting its kinase activity and promoting degradation of the catalytic subunit p110α. This dual mechanism suppresses phosphorylation of the downstream effector AKT, leading to reduced cell proliferation and induction of apoptosis, ultimately exerting antitumor effects. The global, multicenter, double-blind, randomized, Phase III INAVO120 trial evaluated the efficacy and safety of inavolisib combined with palbociclib and fulvestrant versus placebo plus palbociclib and fulvestrant in patients with PIK3CA-mutated HR+/HER2− locally advanced or metastatic breast cancer. At a median follow-up of 21.3 months in the inavolisib group and 21.5 months in the placebo group, the median PFS was 15.0 months (95% confidence interval [CI], 11.3–20.5) versus 7.3 months (95% CI, 5.6–9.3), respectively, corresponding to a hazard ratio for disease progression or death of 0.43 (95% CI, 0.32–0.59; P < 0.001). Based on these findings, in March 2025, the National Medical Products Administration (NMPA) of China approved inavolisib in combination with palbociclib and fulvestrant as a treatment for patients with endocrine therapy-resistant PIK3CA-mutated, HR+/HER2− locally advanced or metastatic breast cancer, including those with recurrence during or after adjuvant endocrine therapy (N. M. P. Administration, 2025). Inavolisib thereby became the first third-generation, highly selective PI3Kα inhibitor approved in China, providing patients with a novel therapeutic option.

An increasing number of studies have sought to evaluate the balance between costs and health benefits of cancer therapies to determine the economic viability of novel agents and treatment regimens. However, pharmacoeconomic research on breast cancer—particularly HR+/HER2− disease—in China remains limited, with most existing studies focusing on agents such as trastuzumab, cisplatin, paclitaxel, and tucatinib (Chen et al., 2025; Deng et al., 2022; Rui et al., 2020; Q. Wu et al., 2020). Although inavolisib has demonstrated superior clinical efficacy, its high cost poses a considerable challenge. At the recommended daily dose of 9 mg, the estimated cost per treatment cycle is approximately USD 4,000, and the prolonged treatment duration imposes a substantial and persistent financial burden on patients. To date, the economic evaluation of inavolisib in HR+/HER2− breast cancer has been incomplete, and its cost-effectiveness requires further investigation. Accordingly, the present study adopts the perspective of the Chinese healthcare system, utilizing clinical data from the INAVO120 trial and applying cost-utility analysis to assess the economic value of inavolisib in HR+/HER2− breast cancer. This evaluation aims to provide an evidence-based economic rationale to inform treatment decision-making and policy implementation.

## 2 Methods

### 2.1 Patients and interventions

This study was conducted in accordance with the consolidated health economic assessment reporting standards (CHEERS) economic evaluation criteria (Supplementary Table S1). The target population and treatment strategies were derived from the INAVO120 clinical trial, a randomized, double-blind, Phase III study. Patients were recruited across 28 countries. Eligible participants were required to have PIK3CA-mutated, HR+/HER2− locally advanced or metastatic breast cancer and could include premenopausal, perimenopausal, or postmenopausal women as well as men. Additional eligibility criteria included disease recurrence or progression within 12 months following adjuvant endocrine therapy or its completion (excluding patients with *de novo* metastatic breast cancer), fasting blood glucose levels below 126 mg/dL, glycated hemoglobin (HbA1c) levels below 6.0%, and measurable disease according to the Response Evaluation Criteria in Solid Tumors (RECIST).

Patients were randomly assigned in a 1:1 ratio to receive either inavolisib (9 mg orally, once daily on days 1–28 of each 28-day cycle) or placebo (once daily), each administered in combination with palbociclib (125 mg orally, once daily on days 1–21 of each 28-day cycle) and fulvestrant (500 mg intramuscularly on days 1 and 15 of cycle 1, and approximately every 28 days thereafter). Randomization was conducted using a block randomization design. Patients who experienced progressive disease (PD) or developed unacceptable adverse events during treatment were allowed to continue therapy with everolimus at a daily oral dose of 10 mg.

### 2.2 Model construction

**FIGURE 1:**
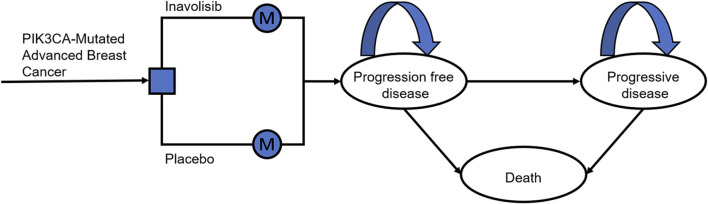
The structure of the partitioned survival model for PIK3CA-mutated advanced breast cancer.

A partitioned survival model was developed comprising three mutually exclusive health states: PFS, PD, and death. This model was employed to simulate and compare the clinical outcomes and economic burdens associated with the two treatment strategies. All patients were assumed to enter the model in the PFS state, with transitions restricted to a unidirectional pathway: PFS → PD → death (Figure 1). Accordingly, patients could only transition forward without the possibility of reverting to a previous health state. For instance, individuals in the PD state could either remain in that state or transition to death, but could not return to the PFS state.

The model cycle length was set at 4 weeks, consistent with the treatment schedule in the INAVO120 trial. The simulation adopted a lifetime horizon and was continued until 99% of the hypothetical cohort had experienced death, thereby ensuring comprehensive estimation of the long-term survival benefits and economic consequences of treatment. The primary outcomes included total costs, quality-adjusted life years (QALYs), and the incremental cost-effectiveness ratio (ICER). Both costs and health outcomes were adjusted on a semi-periodic basis and discounted at an annual rate of 5% in accordance with Chinese health economic evaluation guidelines. The willingness-to-pay (WTP) threshold was defined as USD 40,271 per QALY gained, corresponding to three times the current *per capita* gross domestic product (GDP) of China.

### 2.3 Clinical data input

Due to the unavailability of individual patient data and the limited duration of follow-up, survival curves for PFS and OS from the INAVO120 trial were digitized using GetData Graph Digitizer (version 2.26) to extract survival data (Supplementary Figure S1). Individual-level patient data were then reconstructed in R software (version 4.2.2) and fitted using Royston–Parmar (RP) spline models (Xu et al., 2025). Model selection was based on visual inspection in combination with statistical evaluation using the Akaike information criterion (AIC) and Bayesian information criterion (BIC), with detailed values reported in Supplementary Table S2. Survival curve fitting results under different knot specifications are presented in Supplementary Figure S2, while hazard and cumulative hazard functions are shown in Supplementary Figures S3, S4.

Based on the selected models, survival functions were used to calculate the proportion of patients in each health state at every time point. The proportion in the PFS state was obtained directly from the fitted PFS curve, the proportion of deaths was calculated as 100% minus the OS curve, and the proportion in the PD state was determined as the difference between the OS and PFS curves. The final fitting results are presented in Supplementary Figure S5.

### 2.4 Cost and utility input

This study considered only direct medical costs, including drug acquisition, subsequent therapy, laboratory and imaging examinations, hospitalization, routine follow-up, and management of treatment-emergent adverse events (TEAEs; ≥Grade 3). All costs were converted to US dollars using the exchange rate in September 2025 (1 USD = 7.1329 CNY) for consistency in reporting (China, 2025). Only TEAEs with an incidence of at least 5% and severity grade 3 or higher were incorporated, namely neutropenia, thrombocytopenia, stomatitis, anemia, hyperglycemia, and leukopenia. Drug prices were obtained from publicly available data on the Pharmaceutical Intelligence Network (Zhi, 2025), whereas costs associated with adverse event management were derived from a combination of public databases, local fee schedules, and clinical expert consultation. TEAE incidence rates were sourced from the INAVO120 trial, and other direct medical costs were estimated using public databases and real-world local fee schedules.

Utility values were used to quantify patient preferences for health states, ranging from 0 (death) to 1 (perfect health). As the INAVO120 trial did not systematically collect quality-of-life data, the utility values for PFS and PD were obtained from published literature and set at 0.837 and 0.443 (Jia et al., 2025; Liu et al., 2025; Okeke et al., 2025; Sun et al., 2025; Zhang et al., 2025), respectively. Disutilities associated with grade ≥3 TEAEs occurring at an incidence of at least 5% were also derived from previously published studies (Shu et al., 2025; W. Wu et al., 2023). Key input parameters are summarized in Table 1.

**TABLE 1:** Model parameters and the range of the sensitivity analysis.

Parameters	Baseline value	Range		Distribution
		Minimum	Maximum	
Cost of drugs per cycle, $				
Inavolisib	4065.70	3252.56	4878.84	Gamma
Palbociclib	419.60	335.68	503.52	Gamma
Fulvestrant	646.59	517.27	775.90	Gamma
Everolimus	459.28	367.43	551.14	Gamma
Cost of AEs per cycle, $				
Neutropenia	28.04	22.43	33.65	Gamma
Thrombocytopenia	42.06	33.65	50.47	Gamma
Stomatitis and mucosal inflammation	10.51	8.41	12.62	Gamma
Anemia	21.03	16.82	25.24	Gamma
Hyperglycemia	13.33	10.67	16.00	Gamma
Leukopenia	28.04	22.43	33.65	Gamma
Diarrhea	11.22	8.97	13.46	Gamma
Other costs per cycle, $				
Laboratory tests	59.89	47.91	71.87	Gamma
Radiological examinations	152.95	122.36	183.55	Gamma
Hospitalization and daily care	90.71	72.57	108.85	Gamma
Supportive care	140.20	112.16	168.24	Gamma
Follow-up	11.64	9.31	13.96	Gamma
Utility value				
PFS	0.837	0.670	1.004	Beta
PD	0.443	0.354	0.532	Beta
Neutropenia	0.130	0.104	0.156	Beta
Thrombocytopenia	0.030	0.024	0.036	Beta
Stomatitis and mucosal inflammation	0.220	0.176	0.264	Beta
Anemia	0.070	0.056	0.084	Beta
Hyperglycemia	0.006	0.005	0.007	Beta
Leukopenia	0.130	0.104	0.156	Beta
Diarrhea	0.103	0.082	0.124	Beta
Others				
Discount rate (%)	5	0	8	Beta

AEs, adverse events; PFS, progression-free survival; PD, progressive disease.

### 2.5 Sensitivity analyses

To assess the robustness of the model, both deterministic sensitivity analysis (DSA) and probabilistic sensitivity analysis (PSA) were performed. Owing to the lack of variability data (e.g., standard deviation, standard error, or 95% confidence intervals) for most parameters, DSA was conducted by applying unidirectional or bidirectional variations within predefined ranges. Most parameters were varied by ±20% from their baseline values, whereas parameters with greater uncertainty were varied by up to ±30%. The discount rate was examined across a range of 0%–8% (Liu, 2020). The results of the DSA were illustrated using tornado diagrams.

In the PSA, probability distributions were assigned to model input parameters according to their data characteristics: cost parameters were modeled using a gamma distribution, whereas utility values and probability parameters were modeled using a beta distribution (Briggs et al., 2012). Monte Carlo simulations were then conducted, with each iteration independently sampling all parameter values from their respective distributions. A total of 10,000 iterations were performed to comprehensively assess the joint impact of parameter uncertainty on model outcomes. Results were presented as scatter plots and cost-effectiveness acceptability curves (CEAC).

### 2.6 Scenario analyses

#### 2.6.1 Scenario analysis 1

To improve the accessibility and affordability of novel anticancer therapies, the National Reimbursement Drug List (NRDL) in China has progressively incorporated multiple innovative agents through a dynamic access mechanism. Although inavolisib is not currently included in the NRDL, future price reductions may occur through policy instruments such as national insurance negotiations or centralized bulk procurement. To account for this possibility, we modeled a series of scenarios in which the unit price of inavolisib was reduced incrementally by 5% from the baseline price. The corresponding ICERs were then calculated to evaluate the impact of potential price reductions on cost-effectiveness outcomes.

#### 2.6.2 Scenario analysis 2

The choice of time horizon in a cost-effectiveness model can substantially affect the cumulative estimation of long-term costs and benefits. To explore the impact of this parameter on the robustness of model outcomes, the simulation time horizon was varied across 5, 10, 15, 20 years, and lifetime. The ICERs obtained under each scenario were compared to assess the sensitivity of model results to assumptions regarding the analysis period.

#### 2.6.3 Scenario analysis 3

Given that approximately 60% participants in the INAVO120 trial were from Western countries, we conducted this scenario analysis to test whether alternative health-state utility inputs from non-Chinese cohorts would influence model outcomes. We applied utility values reported in the US HR+/HER2− advanced breast cancer populations (PFS = 0.736; PD = 0.630) to replace the base-case utilities and then recalculated total costs, QALYs, and the ICER.

## 3 Results

### 3.1 Base-case analysis results

The base-case results are shown in Table 2. The total cost of the inavolisib treatment group was higher than that of the placebo group, with costs of USD 194306.06 versus USD 55938.19 In terms of health outcomes, the inavolisib treatment group also performed better, achieving 2.999 QALYs compared with 1.744 QALYs in the placebo group. The incremental cost and QALY for the inavolisib treatment group were USD 138367.86 and 1.255 QALYs, respectively. As a result, the ICER for inavolisib was USD 110260.53/QALY, which exceeded the WTP threshold of USD 40271.00/QALY.

**TABLE 2:** Results of base-case analysis.

Treatment	Total cost, $	Incremental cost, $	QALYs	Incremental QALYs	ICER, $/QALY
Inavolisib	194306.06	138367.86	2.999	1.255	110260.53
Placebo	55938.19	​	1.744	​	​

QALY, quality-adjusted life year; ICER, incremental cost-effectiveness ratio.

### 3.2 Sensitivity analyses results

**FIGURE 2:**
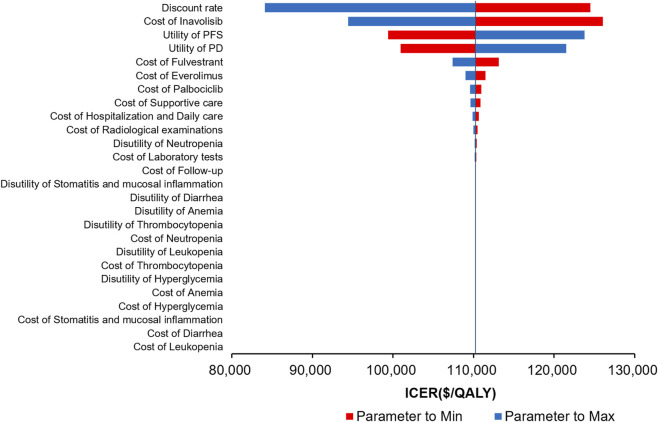
The deterministic sensitivity analyses for inavolisib combined with palbociclib and fulvestrant versus palbociclib and fulvestrant alone. PFS, progression-free survival; PD, progressive disease; ICER, incremental cost-effectiveness ratio; QALY, quality-adjusted life year.

The results of the DSA are presented in the tornado plot (Figure 2). Among all input parameters, the factors with the greatest influence on the ICER were the discount rate and the cost of inavolisib, followed by the utility value for PD and the utility value for PFS; variations in these parameters resulted in considerable fluctuations in the ICER. Parameters with a moderate impact included the costs of fulvestrant, palbociclib, and everolimus, as well as imaging examination fees. Laboratory test costs, hospitalization and routine care expenses, the utility decrement associated with neutropenia, and the disutility of oral mucositis exerted only limited influence on the ICER. Parameters with the least impact were costs associated with managing adverse events such as neutropenia, anemia, and thrombocytopenia, where variations had negligible effects. Overall, across the predefined ranges of parameter variation, the ICER consistently remained above the WTP threshold, demonstrating that the model results were robust.

**FIGURE 3:**
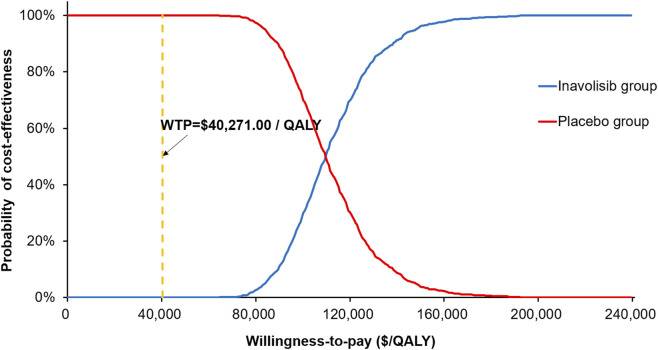
The cost-effectiveness acceptability curves of inavolisib combined with palbociclib and fulvestrant versus palbociclib and fulvestrant alone. WTP, willingness-to-pay; QALY, quality-adjusted life year.

The scatter plot of Monte Carlo simulation results and the CEAC are presented in Figure 3 and Supplementary Figure S6, respectively. The PSA demonstrated that, at the current price of inavolisib, all simulated ICER values were located well above the WTP threshold of USD 40271.00/QALY, indicating that inavolisib is unlikely to be cost-effective in China at the prevailing price level (Supplementary Figure S6). The CEAC further showed that the probability of inavolisib being cost-effective began to increase only when the WTP threshold exceeded USD 100,000/QALY. At a threshold of USD 109,800/QALY, the probability reached 50%, and at USD 138,000/QALY, the probability increased to 90% (Figure 3).

### 3.3 Scenario analysis results

**FIGURE 4:**
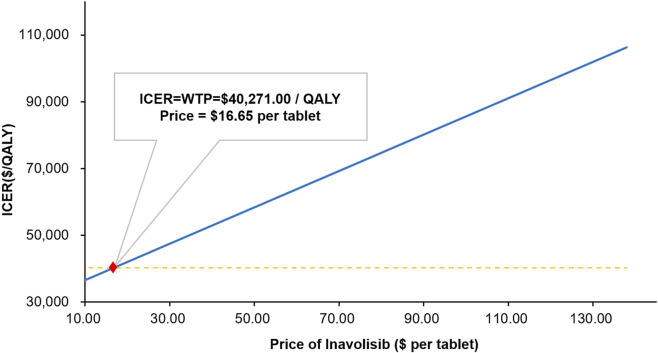
ICER variation with the price of inavolisib. ICER, incremental cost-effectiveness ratio; WTP, willingness-to-pay; QALY, quality-adjusted life year.

The results of Scenario Analysis 1 are summarized in Figure 4 and Supplementary Figure S7. As the unit price of inavolisib decreased, the ICER exhibited a clear downward trend, reflecting progressively improved cost-effectiveness. With a 5% reduction (USD 137.94/tablet), the ICER was USD 106307.75/QALY, far exceeding the WTP threshold. At a 50% reduction (USD 72.60/tablet), the ICER declined to USD 70732.77/QALY, though it remained well above the threshold. When the price decreased by 85% (USD 21.78/tablet), the ICER fell to USD 43063.34/QALY, approaching the WTP threshold. A further reduction of 90% (USD 14.52/tablet) lowered the ICER to USD 39110.56/QALY, falling below the threshold for the first time and indicating that inavolisib would be cost-effective at this price level. More detailed calculations estimated that the break-even price, at which the ICER equaled the WTP threshold, was approximately USD 16.65 per tablet.

The results of Scenario Analysis 2 are presented in Supplementary Table S3. With increasing simulation durations, both total costs and QALYs in the inavolisib treatment group rose over time, while the corresponding ICER showed a consistent downward trend. At 5 years, the inavolisib group incurred total costs of USD 143273.86 and achieved 1.919 QALYs, with incremental costs of USD 98020.69 and incremental QALYs of 0.463, resulting in an ICER of USD 211673.90 per QALY. At 10, 15, and 20 years, the ICER declined to USD 148567.22, USD 127744.87, and USD 119001.60 per QALY, respectively—although all values remained well above the WTP threshold. Overall, the cost-effectiveness of inavolisib improved progressively with longer simulation periods, indicating temporal consistency and stability of the model results.

The result of Scenario Analysis 3 is shown in Supplementary Table S4. The total cost of the inavolisib treatment group was higher than that of the placebo treatment group, with costs of $194306.06 versus $55938.19. In terms of health outcomes, the inavolisib treatment group also performed better, with results of 3.423 QALYs versus 2.006 QALYs. Ultimately, we arrived at ICERs of $97646.36/QALY, consistent with the baseline case result (Line 291–297).

## 4 Discussion

This study evaluated the cost-effectiveness of inavolisib combined with palbociclib and fulvestrant in patients with PIK3CA-mutated, HR+/HER2− locally advanced or metastatic breast cancer, based on data from the INAVO120 Phase III clinical trial. A partitioned survival model was constructed to estimate total costs, QALYs, and ICERs for the inavolisib regimen versus placebo. The base-case analysis showed that the total cost of the inavolisib treatment group was substantially higher than that of the placebo group (USD 194306.06 vs. USD 55938.19), while health outcomes improved markedly (2.999 QALYs vs. 1.744 QALYs). The resulting ICER was USD 110260.53 per QALY, far exceeding China’s reference WTP threshold of USD 40271.00 per QALY, indicating that inavolisib is not cost-effective at its current price.

Sensitivity analyses demonstrated that model outcomes were most sensitive to the discount rate and the cost of inavolisib. The PSA further indicated that the probability of inavolisib being cost-effective in China at current price levels was extremely low. Scenario analyses highlighted that the cost-effectiveness of inavolisib is highly dependent on drug pricing. As the per-tablet price declined, the ICER decreased accordingly, suggesting progressively improved cost-effectiveness. For example, when the price of inavolisib decreased by 90% from the current recommended level (USD 14.52 per tablet), the ICER fell below the WTP threshold for the first time. More detailed calculations estimated that the break-even price—where the ICER equaled the WTP threshold—was approximately USD 16.65 per tablet.

In addition, analysis across different simulation horizons revealed that with longer treatment durations, both total costs and QALYs in the inavolisib group increased, while the ICER gradually declined. These findings suggest that the long-term application of inavolisib may improve cost-effectiveness, although the drug remains economically unfavorable under current pricing conditions.

Previous pharmacoeconomic evaluations for patients with HR+/HER2− locally advanced or metastatic breast cancer (ABC/MBC) have been relatively scarce. Existing studies indicate that CDK4/6 inhibitors combined with letrozole or other endocrine therapies significantly improve PFS and OS (Masurkar et al., 2024); however, the overall incremental net benefit (INB) was −USD 149266.87, failing to meet cost-effectiveness criteria. Jia et al. (2025) evaluated the efficacy, safety, and cost-effectiveness of CDK4/6 inhibitors in second-line treatment for HR+/HER2− ABC/MBC. Results indicated that ribociclib or abemaciclib combined with fulvestrant demonstrated greater cost-effectiveness compared with palbociclib combined with fulvestrant. Zeng et al. (2023) conducted a network meta-analysis and cost-effectiveness evaluation of three first-line CDK4/6 inhibitors (Abemaciclib, Palbociclib, Ribociclib) combined with non-steroidal aromatase inhibitors (NSAI) from a Chinese payer perspective. The results indicated that all three regimens significantly improved PFS and OS. Among them, abemaciclib combined with NSAI demonstrated economic viability at current drug prices, with drug pricing being a key factor influencing cost-effectiveness. These findings align with the present study, further highlighting that drug pricing is a critical determinant of cost-effectiveness.

Although inavolisib is currently associated with a high price in China, with its calculated ICER far exceeding the WTP threshold, its significant clinical benefits are undeniable. With an extended median follow-up of 34.2 months (Jhaveri et al., 2025), the inavolisib group achieved an OS of 34 months compared with 27 months in the placebo group, corresponding to a 33% reduction in the risk of death (HR 0.67; 95% CI, 0.48–0.94; P = 0.019). Taken together with the findings from the baseline and sensitivity analyses of this study, drug pricing emerges as a critical determinant of cost-effectiveness. Reasonable price adjustments and healthcare reimbursement negotiations could substantially improve the economic profile of inavolisib.

Past experience provides encouraging evidence. During the 2024 National Healthcare Insurance negotiations (N. H. S. Administration, 2024a), nearly 90 new drugs were included, of which 26 were oncology agents, achieving an average price reduction of 67%. Likewise, the 10th round of centralized procurement in 2025 included several anticancer drugs (N. H. S. Administration, 2024b), all of which underwent substantial price reductions. For example, the price of liposomal doxorubicin hydrochloride injection dropped from approximately ¥3,000–4,550 per vial to as low as ¥98 following procurement. For breast cancer patients requiring around 13 doses annually (40 mg per dose, equivalent to 2 vials), the annual cost decreased from nearly ¥78,000 to ¥2,548, representing a reduction of more than 90%. Given the substantial clinical benefit of inavolisib, similar price reductions could be expected if the drug were included in the National Reimbursement Drug List. Such measures would significantly enhance its affordability and accessibility, providing a more viable treatment option for patients with HR+/HER2− breast cancer in China.

Additionally, regional disparities in economic development and patient affordability substantially influence drug accessibility. In high-GDP regions such as Shanghai and Beijing, where patients have greater purchasing power, inavolisib may demonstrate a higher probability of cost-effectiveness even at elevated prices. Conversely, in economically less developed areas, where patients are more price-sensitive, strategies such as price reductions or drug donation programs are necessary to enhance accessibility.

In the extended scenario analysis (Supplementary Figure S8), when the price of inavolisib was reduced to USD 47.92 per tablet (a 67% decrease from the original price), the CEAC curve shifted leftward overall. Although the ICER after price reduction (USD 57293.33 per QALY) remained above the national WTP threshold, the regimen demonstrated economic viability in economically developed regions. Using three times the 2024 provincial GDP *per capita* to estimate region-specific WTP thresholds, Beijing (USD 95898.55 per QALY) and Shanghai (USD 91198.89 per QALY) substantially exceeded the national average, and under this pricing scenario their cost-effectiveness acceptance probability reached 100%. In contrast, regions with intermediate economic levels such as Zhejiang (USD 57202.66 per QALY) had lower thresholds, corresponding to a cost-effectiveness probability of approximately 50.80%. Further price reductions or risk-sharing payment models remain necessary to reduce pressure on medical insurance funds. In less developed regions such as Gansu (USD 22186.50 per QALY), the WTP threshold was significantly below the national average; even with a 67% price reduction, the ICER of inavolisib remained far above the threshold, resulting in a near-zero cost-effectiveness probability.

This study has several limitations. First, the model was constructed using data from the INAVO120 clinical trial to simulate real-world patient characteristics and treatment pathways. However, the results relied on multiple input parameters and assumptions (e.g., utility values, cost data, discount rates), some of which were derived from published literature, thereby introducing inherent uncertainty. Second, due to the relatively short follow-up period, long-term survival outcomes required extrapolation. Although individual patient data were reconstructed from Kaplan–Meier curves and multiple parametric models were tested to identify the best-fitting distribution, extrapolated long-term estimates inevitably carry some degree of bias. Third, for the sake of simplicity, only treatment-related adverse events of grade ≥3 with an incidence of at least 5% were included in the model, which may underestimate the total cost associated with toxicities. Nevertheless, sensitivity analyses suggested that the costs of adverse events had only a limited impact on the overall model outcomes. The last limitation of this study is the lack of Asian subgroup survival data from the INAVO120 trial. Although the trial reported overall hazard ratios for the intention-to-treat population, detailed progression-free survival or overall survival estimates for Asian patients, including Kaplan–Meier curves or subgroup-specific HRs, were not publicly available. Because these data are essential inputs for survival modeling, a formal subgroup analysis could not be performed. As a result, the present evaluation relied on the global HRs as proxies for the Chinese population. This assumption may introduce bias, as treatment response, pharmacogenomic characteristics, and clinical practice patterns may differ between Asian and Western populations. Moreover, this study simplified post-progression therapy by assuming no treatment switching and applying a single representative regimen (fulvestrant plus everolimus) for all patients in the PD health state. This structural simplification was necessary because standardized downstream treatment sequences after first-line PI3K inhibitor–based therapy are not yet established. However, real-world clinical management is often heterogeneous, with treatment selection influenced by progression dynamics, visceral involvement, and endocrine sensitivity. If a substantial proportion of patients receive alternative therapies after progression, both total costs and QALYs may diverge from our model estimates. Future evaluations incorporating real-world treatment patterns and post-progression sequencing data would provide a more comprehensive assessment of the economic value of inavolisib-based strategies.

## 5 Conclusion

The results of this study indicate that, compared with palbociclib and fulvestrant monotherapy, the addition of inavolisib significantly prolonged PFS and OS in patients with PIK3CA-mutated, HR+/HER2− advanced breast cancer. However, under current drug pricing, the ICER for this regimen was USD 110260.53 per QALY, far exceeding the WTP threshold of USD 40271.00 per QALY, and therefore lacking cost-effectiveness. Scenario analysis suggested that the regimen would become economically viable only if the drug price were reduced by approximately 88.53%. Overall, these findings provide important evidence to inform clinical treatment decision-making and health insurance reimbursement policy for patients with advanced breast cancer.

## Data Availability

The original contributions presented in the study are included in the article/Supplementary Material, further inquiries can be directed to the corresponding author.
